# Fragile X checklists: A meta‐analysis and development of a simplified universal clinical checklist

**DOI:** 10.1002/mgg3.398

**Published:** 2018-04-06

**Authors:** Toni Kasole Lubala, Aimé Lumaka, Gray Kanteng, Léon Mutesa, Olivier Mukuku, Stanislas Wembonyama, Randi Hagerman, Oscar Numbi Luboya, Prosper Lukusa Tshilobo

**Affiliations:** ^1^ Division of Dysmorphology & Birth Defects Department of Pediatrics University of Lubumbashi Lubumbashi Congo; ^2^ Faculté de Médecine Département de Pédiatrie Université de Kinshasa Kinshasa Congo; ^3^ Centre de Génétique Humaine Institut National de Recherche Biomédicale Kinshasa Congo; ^4^ Center for Human Genetics School of Medicine and Pharmacy College of Medicine and Health Sciences University of Rwanda Kigali Rwanda; ^5^ Département de Pédiatrie Institut Supérieur des Techniques Médicales Lubumbashi Congo; ^6^ MIND Institute University of California Davis Sacramento CA USA; ^7^ Department of Pediatrics University of California Davis Medical Center Sacramento CA USA

**Keywords:** checklists, clinical features, fragile X, meta‐analysis

## Abstract

**Background:**

Clinical checklists available have been developed to assess the risk of a positive Fragile X syndrome but they include relatively small sample sizes. Therefore, we carried out a meta‐analysis that included statistical pooling of study results to obtain accurate figures on the prevalence of clinical predictors of Fragile X syndrome among patients with intellectual disability, thereby helping health professionals to improve their referrals for Fragile X testing.

**Methods:**

All published studies consisting of cytogenetic and/or molecular screening for fragile X syndrome among patients with intellectual disability, were eligible for the meta‐analysis. All patients enrolled in clinical checklists trials of Fragile X syndrome were eligible for this review, with no exclusion based on ethnicity or age. Odds ratio values, with 95% confidence intervals as well as Cronbach coefficient alpha, was reported to assess the frequency of clinical characteristics in subjects with intellectual disability with and without the fragile X mutation to determine the most discriminating.

**Results:**

The following features were strongly associated with Fragile X syndrome: skin soft and velvety on the palms with redundancy of skin on the dorsum of hand [OR: 16.85 (95% CI 10.4–27.3; α:0.97)], large testes [OR: 7.14 (95% CI 5.53–9.22; α: 0.80)], large and prominent ears [OR: 18.62 (95% CI 14.38–24.1; α: 0.98)], pale blue eyes [OR: 8.97 (95% CI 4.75–16.97; α: 0.83)], family history of intellectual disability [OR: 3.43 (95% CI 2.76–4.27; α: 0.81)] as well as autistic‐like behavior [OR: 3.08 (95% CI 2.48–3.83; α: 0.77)], Flat feet [OR: 11.53 (95% CI 6.79–19.56; α:0.91)], plantar crease [OR: 3.74 (95% CI 2.67–5.24; α: 0.70)]. We noted a weaker positive association between transverse palmar crease [OR: 2.68 (95% CI 1.70–4.18; α: 0.51)], elongated face [OR: 3.69 (95% CI 2.84–4.81; α: 0.63)]; hyperextensible metacarpo‐phalangeal joints [OR: 2.68 (95% CI 2.15–3.34; α: 0.57)] and the Fragile X syndrome.

**Conclusion:**

This study has identified the highest risk features for patients with Fragile X syndrome that have been used to design a universal clinical checklist.

## 1 INTRODUCTION

Fragile X Syndrome (FXS) is the most common known inherited form of intellectual disability and has been reported as the most common known inherited single‐gene disorder associated with autism spectrum disorder (ASD), accounting for 2%–3% of all cases of ASD (Sherman et al., 1985). Studies estimate the prevalence of FXS to be 1 in 4,000 to 1 in 7,000 in males and 1 in 6,000 to 1 in 11,000 in females (Crawford et al., 2002). In 99% of patients, the molecular basis of the FXS is an expanded CGG repeat string (>200 hyper methylated CGG repeats, full mutation) in the 5′ untranslated region of the *FMR1* gene located at Xq27.3. Since the identification of FXS as a major cause of intellectual disability (ID), extensive screening programs have been developed and carried out to identify patients in many countries. To increase the efficiency of the screening programs, about 10 clinical checklists have been developed for preselection of subjects based on clinical features. The use of checklists to select patients with a high probability of being affected by FXS may significantly reduce the number of individuals to be submitted to molecular evaluation (Mandel & Chelly, 2004), greatly improving the cost‐effectiveness of Fragile X testing. Such clinical checklists have been developed in different populations with different ethnic backgrounds such as Caucasians, African Americans, Latinos, Indians, and Chinese (Butler, Mangrum, Gupta, & Singh, 1991; Giangreco, Steele, Aston, Cummins, & Wenger, 1996; Guo et al., 2000; Hagerman, Amiri, & Cronister, 1991; Laing, Partington, Robinson, & Turner, 1991; Limprasert et al., 2000; Maes, Fryns, Ghesquiere, & Borghgraef, 2000).

In our recent paper, we shown that facial dysmorphism is influenced by ethnic background of the patient (Lumaka et al., 2017). Moreover, clinical available checklists have been developed in relatively small sample sizes, increasing the chance of assuming as true a false hypothesis. Therefore, we carried out a meta‐analysis that included statistical pooling of study results to obtain accurate figures on the prevalence of clinical predictors of FXS among patients with ID. This meta‐analysis also aims to assess variations in prevalence of clinical features among patients from different ethnic backgrounds, thereby helping to develop a universal clinical checklist to improve their referrals for Fragile X testing.

## 2 MATERIALS AND METHODS

### 2.1 Assessment of studies for inclusion in this review

Two coauthors independently conducted a systematic review of previous published studies for inclusion in the present work. Included studies were assessed based on trial quality. Data were extracted independently, and a meta‐analysis was performed after transforming reported data using the intention‐to screen principle. This review used standard methods proposed by the Cochrane Collaboration (van Tulder, Furlan, Bombardier, & Bouter, 2003).

### 2.2 Types of studies

All published studies consisting of cytogenetic and/or molecular screening for fragile X syndrome among patients with intellectual disability, were eligible for the meta‐analysis.

### 2.3 Types of participants

All patients enrolled in clinical checklists studies of FXS were eligible for this review, with no exclusion based on ethnicity or age.

### 2.4 Search strategy for identification of studies

Electronic searches of the specific journals and technical reports were performed.

### 2.5 Methods of the review

Two authors independently selected studies for possible inclusion against a predetermined checklist of inclusion criteria.

We searched in PubMed and Google Scholar from January 1991 to December 2016 for articles in English, German, French, Spanish, Portuguese and Chinese. Following keywords/terms were searched: Fragile X syndrome*, checklist*, screening*, signs*, and diagnosis*. An asterisk after a term means that all terms that begin with that root were included in the search.

#### 2.5.1 Step 1

Abstracts were reviewed by the first author (TL) and selected for further review if they met one of the following two criteria: (1) Significant studies that covered Fragile X syndrome clinical features (2) Clinical checklists for FXS screening. If a criterion was not met because not enough information was provided, the abstract was set aside for further evaluation.

#### 2.5.2 Step 2

Abstracts were reviewed independently by two authors (TL and GK) and were selected based on their consensus according to the same criteria used in Step 1. If consensus was not reached, the abstract was then set aside for further evaluation.

#### 2.5.3 Step 3

Full‐text articles of abstracts selected in Step 2 were retrieved and reviewed by one author (TL). Inclusion was based on consensus between two investigators (TL and GK). Disagreements were discussed with a third author (OL). Studies were included if they met the following criteria: (1) Clinical checklists development studies and (2) diagnosis based on molecular analysis of the Fragile X mutation. The exclusion criteria were (1) studies that did not report confidence intervals (CI) or standard errors (SE) and that (2) did not report data that allowed the calculation of these parameters and whose authors did not provide such data upon request.

### 2.6 Assessment of quality

Two authors independently assessed the susceptibility to bias of the selected articles. The risk of bias was assessed by reporting the study's conduct against the criteria described in the Cochrane Handbook for Systematic Reviews of Interventions (van Tulder et al., 2003). Studies were categorized as attributing a ‘low’, ‘moderate’, or ‘high’ risk of bias.

### 2.7 Data extraction

Data were independently extracted by two coauthors using a standard data extraction form. We extracted the following data from each study: region(s) in which the study was conducted, age group, gender, ethnicity of study sample, year when the study was conducted, and dysmorphic features as well as behavioral phenotype such as Autistic‐like behavior (Tactile defensiveness, perseverative speech, hand flapping, poor eye contact) and attention‐deficit hyperactivity disorders (ADHD) in subjects with ID with and without the Fragile X mutation. We classified region(s) in which the study was conducted according to the United Nations’ classification of macro‐geographic continental regions, namely, Asia, Africa, Europe, North America, Latin America and the Caribbean, and Oceania. Disagreements between coauthors were rare. In the event of a disagreement the data were reviewed and resolved by discussion by three authors.

### 2.8 Statistical analysis

Odds ratio (OR) values, with 95% confidence intervals (CI), were reported to assess the frequency of clinical characteristics in subjects with ID with and without the Fragile X mutation to determine the most discriminating. Between‐study heterogeneity was estimated using the χ^2^‐based Q statistic. Heterogeneity was considered statistically significant when *p*_heterogeneity_ <0.1. A statistical test with a *p*‐value less than .05 was considered significant. All analyses were performed with RevMan software (version 5.1 for Windows, The Cochrane Collaboration, Copenhagen, Denmark). A random‐effect meta‐analyses model have been chosen to assume that the observed clinical feature prevalence can vary across studies because of differences in study populations (age, ethnicity). To confirm the associations found in the meta‐analysis, we used the reliability test by calculating the Cronbach coefficient. Found factors with values between 0.91 and 1.0 gave a strong association with FXS and those with values between 0.70 and 0.90 showed a good association.

The X‐fragile diagnosis score is calculated using an index built from the patient's signs. (See the table above).

The index is built as follows:

- Each sign is rated 1 or 2.
- The score obtained are standardized according to a normal standardized distribution. Mean = 0; standard deviation = 1.
- We attribute to each patient a final score which result from the sum of the ratings of each sign present.
- Each patient is classified according to the score obtained.

The interpretation of the score (Min = 1, Max = 10).

≥5: X‐fragile diagnosis is certain.

<5: X‐fragile diagnosis is negative.

## 3 RESULTS

**Figure 1:**
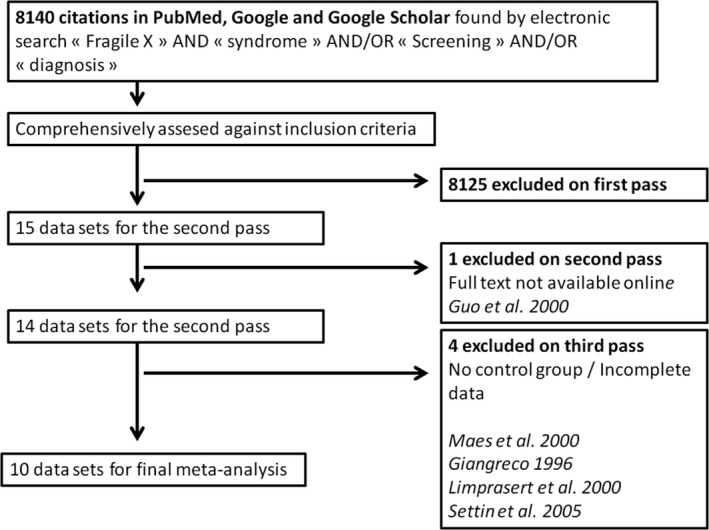
Flow diagram of search strategy and study selection

Of the initial 8,140 records, two reviewers determined independently that 16 required a full review of the manuscript. The selection process of studies for inclusion in the meta‐analysis is shown in Figure 1. Six studies have been excluded after the second and the third pass (Giangreco et al., 1996; Guo et al., 2000; Limprasert et al., 2000; Maes et al., 2000; Settin, Al‐Haggar, Al‐Baz, Al‐Aiouty, & Hafez, 2005). Our final primary analysis included 10 articles and the findings are summarized below in the tables. All papers used in our analysis were published in English, except for one that was written in Portuguese.

Table 1 summarizes the characteristics of included studies. It shows that six studies were from North America and Europe (Arvio, Peippo, & Simola, 1997; Bellavance & Morin, 2017; Butler, Brunschwig, Miller, & Hagerman, 1992; Hagerman et al., 1991; Lachiewicz, Dawson, & Spiridigliozzi, 2000; de Vries, Halley, Oostra, & Niermeijer, 1998), two from South to Central Asia (Guruju et al., 2009; Kanwal et al., 2015), two from Latin America and the Caribbean (Boy, Correia, Llerena, Machado‐Ferreira, & Pimentel, 2001; Christofolini et al., 2009), and one from Africa (Behery, 2008). We did come across two interesting fragile X syndrome studies from Sub‐Saharan Africa, but none of them met the criteria to be included in this study (Essop & Krause, 2013; Peprah, Allen, Williams, Woodard, & Sherman, 2010). One American cohort was multiethnic, including African‐American patients.

**Table 1:** Baseline characteristics for studies included in meta‐analysis

Study	Year	Sample size *n* (%)				Age (Mean)	Gender ratio (M:F)	Method for Genetic diagnosis of FXS	Population ethnicity	
		*n*	X Fra +	X Fra −	*p*					
Buttler et al.	1991	188	19 (10.1)	169 (89.9)	˂.001	3.7‐71.9 (21.3)	1:0	CG	North America (USA)	
Hagerman et al.	1991	106	15 (14.2)	91 (85.8)	˂.001	1‐58 (30 ± 5)	1:0	CG	North America (USA)	
Arvio et al.	1997	370	26 (7.0)	344 (93.0)	˂.001	21‐54 (31.7 ± 11)	1:0	CG	Europ (Finland)	
De Vries et al.	1998	896	9 (1.0)	887 (99.1)	˂.001	NA (NA)	1:0	PCR (SB)	Europ (Netherland)	
Lachiewizc et al.	2000	73	36 (49.3)	37 (50.7)	.99	NA (6.2 ± 2.4)	1:0	CG, DNA	North America (USA)	
Boy et al.	2001	92	14 (15.2)	78 (84.8)	˂.001	7‐27 (13.4)	5:1	CG	Latin America and the Caribbean (Brazil)	
Behery	2008	200	34 (17.0)	166 (83.0)	˂.001	2–20 (NA)	1:0	RT‐PCR	Africa (Egypt)	
Christofolini et al.	2009	192	30 (15.6)	162 (84.4)	˂.001	2–31 (11.3 ± 5.6)	1:0	PCR (SB)	Latin America and the Caribbean (Brazil)	
Guruju et al.	2009	327	25 (7.7)	302 (92.3)	˂.001	4–16 (NA)	1:0	PCR (SB)	Asia (India)	
Kanwal et al.	2015	357	13 (3.6)	344 (96.4)	˂.001	4–40 (14.28 ± 7.01)	7:3	PCR (SB)	Asia (Pakistan)	

CG, Cytogenetics; SB, Southern Blot; RT‐PCR, Real time Polymerase Chain Reaction.

Table 2 shows the results of meta‐analyses of Clinical features in Fragile X positive and in Fragile X negative patients. Skin soft and velvety on the palms with redundancy of skin on the dorsum of hand [Odds ratio: 16.85 (95% CI 10.4–27.3; α:0.97)], large testes [Odds ratio: 7.14 (95% CI 5.53–9.22; α: 0.80)], large and prominent ears [Odds ratio: 18.62 (95% CI 14.38–24.1; α: 0.98)], pale blue eyes [Odds ratio: 8.97 (95% CI 4.75–16.97; α: 0.83)], family history of intellectual disability [Odds ratio: 3.43 (95% CI 2.76–4.27; α: 0.81)] as well as autistic‐like behavior [Odds ratio: 3.08 (95% CI 2.48–3.83; α: 0.77)], flat feet [Odds ratio: 11.53 (95% CI 6.79–19.56; α:0.91)], plantar crease [Odds ratio: 3.74 (95% CI 2.67–5.24; α: 0.70)] were strongly associated with the fragile X syndrome. We noted a weaker positive association between Transverse palmar crease [Odds ratio: 2.68 (95% CI 1.70–4.18; α: 0, 51)], Elongated face [Odds ratio: 3.69 (95% CI 2.84–4.81; α: 0.63)]; hyperextensible metacarpo‐phalangeal joints [Odds ratio: 2.68 (95% CI 2.15–3.34; α: 0.57)] and the fragile X syndrome.

**Table 2:** Pooled prevalence and Odds ratio of Physical, Behavioral, and cognitive traits in patients with intellectual disability with and without fragile X syndrome

Traits	X Frag +	X Frag −	Odds ratio random (IC95%)	Heterogeneity			Test for overall effect		Cronbach Coeff.
				χ^2^	*df* (*p*)	*I* ^2^	*z*	*p*	α
Elongated face	109/151	533/2728	3.69 (2.84‐4.81)	638.6	18 (˂.001)	45	103.9	˂.001	0.63
Hyperextensible metacarpo‐phalangeal joints	150/220	849/3336	2.68 (2.15‐3.34)	492.6	24 (˂.001)	37	80.3	˂.001	0.57
Skin soft and velvety	38/43	95/1811	16.85 (10.4‐27.3)	512.8	3 (˂.001)	57	212.1	˂.001	***0.97***
Flat feets	26/37	43/115	11.53 (6.79‐19.56)	46.4	3 (.001)	41	3.5	.061	***0.91***
Transverse palmarcrease/sydney lines	30/115	104/1064	2.68 (1.70‐4.18)	45.2	12 (.012)	18	18.3	˂.001	0.51
Plantar crease	84/98	162/707	3.74 (2.67‐5.24)	232.8	9 (.001)	42	62.0	˂.001	***0.70***
Large and prominent ears	173/206	756/3458	18.62 (14.38‐24.1)	604.3	24 (.001)	39	163.7	˂.001	***0.98***
Large testicles	129/181	291/2915	7.14 (5.53‐9.22)	290.5	18 (.001)	33	281.4	˂.001	***0.80***
Pale blue eyes	28/49	23/317	8.97 (4.75‐16.97)	23.6	3 (.002)	24	48.5	˂.001	***0.83***
Tactilely defensive	108/166	626/3274	3.40 (2.63‐4.40)	957.7	24 (.012)	48	94.8	˂.001	0.68
Hand flapping	75/128	404/1391	2.91 (2.20‐3.84)	226.8	15 (.025)	32	20.1	˂.001	0.59
Hand‐biting	45/115	218/1062	1.91 (1.31‐2.77)	84.3	12 (.256)	23	11.0	.001	0.39
Perseverative speech	107/161	675/1466	1.44 (1.11‐1.87)	179.3	15 (.298)	29	7.3	.007	0.31
Poor eye contact	139/161	517/1506	2.51 (1.96‐3.22)	361.4	21 (.012)	37	54.6	˂.001	0.45
Hyperactivity	120/162	829/1576	1.41 (1.10‐1.81)	372.2	18 (.001)	35	6.9	.009	0.41
Familial history of intellectual disability	166/205	807/3418	3.43 (2.76‐4.27)	584.6	24 (˂.001)	84	19.4	˂.001	*0.81*
Short attention	91/115	511/1063	1.65 (1.23‐2.21)	219.0	12 (.458)	33	10.6	.001	0.23
ADHD	122/162	870/1576	1.36 (1.06‐1.75)	510.1	18 (.001)	41	5.7	.017	0.48
Autist Like Behavior^a^	162/213	854/3457	3.08 (2.48‐3.83)	556.4	30 (˂.001)	79	75.6	˂.001	***0.77***

a Tactile defensiveness, perseverative speech, hand flapping, and poor eye contact.

We propose in Table 3 a new checklist with the seven most significant characteristics based on the findings shows on Table 2.

**Table 3:** Clinical scoring for the seven most discriminant fragile X features

Traits	Score	
	1	2
Skin soft and velvety on the palms with redundancy of skin on the dorsum of hand		X
Flat feet		X
Large and prominent ears		X
Plantar crease	X	
Large testicles^a^	X	
Familial history of ID	X	
Autistic‐Like Behavior	X	
Total	4	6

a Postpubertal males only.

## 4 DISCUSSION

Here, we have reviewed in detail 10 articles that had controlled and pertinent data for use in screening populations of individuals with ID or ASD to determine who should receive DNA testing for FXS. Such checklists are important in areas where not everyone with ID or ASD can undergo testing because of limited resources. This analysis has revealed a group of characteristics that could be included in universal screening for FXS. As shown in Table 2, the most discriminating items include large or prominent ears, flat feet and soft velvet like skin and plantar creases. All these features relate to the connective tissue problems involving elastin (poorly developed, disorganized, demonstrating a lack of the elastin tree‐like structure in the dermis) that are present in individuals with FXS (Davids, Hagerman, & Eilert, 1990).

We have identified that the blue eyes are related to FXS. However, this feature is associated with the ethnic background. Even for Caucasians, it just reflects the ethnic bias of about two studies and therefore would be inappropriate for a universal checklist.

Macroorchidism is related to the age and gender of the patient. Therefore, for prepubertal or female children, this item is less appropriate. If pubertal or older male patients are included in the screening, then the growth abnormalities leading to macroorchidism is a helpful feature for clinically identifying those with FXS.

Although some autistic features are more common than others, hand flapping, hand biting, and poor eye contact are seen in the majority of subjects with FXS (Hagerman et al., 1991). For simplification purpose, autistic‐like behaviors have been grouped. This feature is scored as positive when one of the following behaviors is present: tactile defensiveness, hand flapping, hand biting, delayed or perseverative speech, and poor eye contact.

Also important is a family history of ID or ASD since FXS represents about 30% of the causes of X‐linked ID and, in most cases, there is a family history. The clinician can also ask about a family history of premutation problems such as early menopause, because the Fragile X‐associated primary ovarian insufficiency (FXPOI) occurs in about 20% of the carriers, or a history of tremor, ataxia, or cognitive decline, because the Fragile X‐associated tremor ataxia (FXTAS) can occur in about 50% of older male carriers and 16% of older female carriers (Hagerman & Hagerman, 2013).

The main goal for our study was to develop a universal and simplified clinical checklist for FXS screening among subjects with ID based on the results of a meta‐analysis of previous screening studies. In the light of our results, we propose a clinical FXS checklist for any population comprising the following seven items: soft skin and velvety, flat feet, large/prominent ears, family history of ID, plantar crease, autistic‐like behavior, and macroorchidism. We considered the attribution to be of value 2 if soft skin and velvety, flat feet, and large ears are present and 0 if absent. If family history of ID, plantar crease, autistic‐like behavior, and macroorchidism are present, we considered the attribution to be of value 1 and 0 if absent. The maximum score is 10 points for postpubertal male subjects and nine for prepubertal males or female subjects. Patients with score higher than 5 have a significant yield of FXS and thus should be considered for molecular testing to rule out the presence of the Fragile X mutation. This combination of behaviors, family history of ID/ASD, and physical manifestations in the clinical checklist yield a high percentage of subjects with FXS regardless their age, gender, or ethnic background.

For screening programs, we need to have a clinical checklist with 100% sensitivity and the highest possible specificity. Specificity and sensibility of our checklist could not be assessed due to the method of our study. This is the main limitation of our study.

Further validation studies should be undertaken in populations of different ethnic backgrounds to analyze the scores obtained for patients with and without the Fragile X gene mutation, based on the seven items considered to be more discriminant for FXS in the present meta‐analysis.

## 5 CONCLUSION

In general, it is important for all physicians to carry out Fragile X DNA testing for those with ID or ASD and to carry out cascade testing in families where the Fragile X mutation has been identified. In countries where such testing is difficult to obtain, then the testing of those at highest risk for FXS is worthwhile, and this study has identified the highest risk features for patients with FXS that have been used to design a simplified universal clinical checklist.

## ETHICS APPROVAL

All the studies mentioned in this review have been approved by the Ethics Committee.

## CONFLICT OF INTEREST

We declare that we have no conflict of interest.

## AUTHOR CONTRIBUTORS

Toni Lubala and Gray Kanteng contributed to protocol design, search, data extraction, quality assessment, statistical analysis, and writing the report. Aimé Lumaka, Léon Mutesa, Randi Hagermann, Stanis Wembonyama, Oscar Luboya, and Prosper Lukusa‐Tshilobo contributed to writing and revision of the report. TL and RH contributed to interpretation of data and revision of the report. All authors have seen and approved the final version.
